# Factor structure of the Chinese version of the geriatric anxiety inventory

**DOI:** 10.1186/s12991-016-0092-4

**Published:** 2016-01-28

**Authors:** Ming Guan

**Affiliations:** Family Issues Center, Xuchang University, Xuchang, China; School of Business, Xuchang University, Xuchang, China

**Keywords:** Factor structure, GAI-CV, Principal components analysis, Confirmatory factor analysis

## Abstract

**Background:**

As China’s population ages, the mental health of older people has been increasingly focused on by academic circles.

**Purpose:**

The aim of this study was to identify the factor structure of the Chinese version of the geriatric anxiety inventory (GAI-CV).

**Methods:**

This study used data collected from Investigation on the anxiety symptoms of the elderly in the city of Beijing supported by scientific research fund project of Renmin University of China. Cronbach’s α was used to test internal consistency reliability. Both confirmatory and exploratory factor analyses were performed separately for factor analysis.

**Results:**

1318 subjects with mean age 71.35 ± 7.44 years (male 40.6 %) were involved. Principal components analysis revealed a three-factor structure of the GAI-CV. GAI-CV scales exhibited good internal consistency (Cronbach’s α = 0.937) and a three-factor model fit the data well [comparative fit index (CFI) = 0.891, root mean square error of approximation (RMSEA) = 0.084].

**Conclusions:**

The Chinese version of the GAI appears to be reliable and valid to measure anxiety for elderly people in China.

## Background

Late-life anxiety disorders are chronic and fairly common, but under-diagnosed and undertreated. Anxiety disorders in the elderly are an underestimated cause of distress, disability, and mortality risk [1]. The Generalized Anxiety Inventory (GAI) is a new 20-item self-report or nurse-administered scale developed by Pachana et al. (2007) that measures dimensional anxiety in elderly people [2]. Various versions of GAI, such as Australian version [3], Portuguese version [4], Spanish version [5], GAI-short form [6], Chinese version [7], and Brazilian Portuguese version [8] have demonstrated very good psychometric properties and can be a reliable instrument to measure anxiety in native elderly people. The concurrent validity [9] and predictive validity [10] of the GAI were validated. And GAI in a duloxetine clinical trial for elderly adults with generalized anxiety disorder was validated [11].

With the fast development of its economy, China has seen the fastest increase in its aging population in the world. The mental health of life of older people is of increasing interest in China, due to its aging population. But there is lack of systematic evidences of aged Chinese with anxiety. While, worst of all, middle-aged persons suffering from anxiety disorder in modern China live poor lives. For example, Wang et al. (2015) indicate that patients with anxiety disorder in China tend to have poor family functioning and quality of life [12]. Hence, I speculate that elderly Chinese populations are prone to expose themselves to anxiety and be tortured by it.

Anxiety is a common mental disorder among older people who live in the city of Beijing [13]. A study in the Xicheng district of Beijing in China shows that anxiety and gloomy mentality are common in old people aged 60–80 years, which is independently associated with related factors such as life quality, subjective well-being, social support, and so on [14].

The aim of the current preliminary study was to analyze the factor structure of GAI-CV, both with exploratory and confirmatory methods, and also to provide relevant support for methodological decisions.

## Methods

### Data source

Investigation on the anxiety symptoms of the elderly in the city of Beijing used here was supported by scientific research fund project of Renmin University of China in December 2009. In the case of translation, GAI was translated into Chinese language. The Investigation began from September 2010 and ended in February 2012. All the 60 and above subjects were 1350 community elders from 45 villages and residential communities and 101 institutional elders from Sijiqing Homes For The Elderly and Jia De The Aged Apartment in Beijing. The main demographic variables are age, sex, educational level, hukou, and marital status.

### Statistical analysis

In the first stage, descriptive statistics with socioeconomic indicators and exploratory factor analysis (principal component analysis) with varimax rotation (eigenvalues of greater than one) was carried out with SPSS version 16.0 for Windows (SPSS Inc., Chicago, IL, USA). I next studied item loading on each factor after rotation, considering 0.5 as significant loading. Item lodgings of greater than 0.5 were considered as contributing significantly to a particular factor. Cronbach’s α was calculated to measure reliability of each subscale of the GAI-CV represented by the identified factors.

Then I performed a confirmatory factor analysis using the China structure of the GAI using Stata 12.0 (Stata Corporation, Texas, USA). Eight indexes were used to measure goodness of fit: Tucker–Lewis coefficient (TLI), comparative fit index (CFI), root mean square error of approximation (RMSEA), Akaike information index (AIC), Bayesian information criterion (BIC), Standardized root mean squared residual (SRMR), Coefficient of determination (CD), and degree of freedom (*df*). CFI assesses fit relative to a null model and ranges from 0 to 1 with values of 0.90–0.95 indicating acceptable and over 0.95 good fit. TLI adjusts for the number of model parameters and is interpreted as CFI. RMSEA expresses the lack of fit per degree of freedom of the model. Values are interpreted as follows: <0.05 indicates very good, >0.05–0.08 good, and >0.10 poor fit. SRMR is the average of the differences between the observed and predicted correlations and has a range from 0 to 1. Values of <0.08 indicate good fit.

Construct validity is tested using exploratory and confirmatory factor analysis. In this study, reliability was tested using Cronbach’s α to assess the internal consistency of the instruments.

## Results

### Sample characteristics

Participants in this study included 1318 people more than 60 years old who were living in the community. They were randomly selected from 15 communities in Beijing. Basic information was collected in Table 1. Table 1 shows most of subjects were females with education levels less than college level. Regarding ethnicity, only 3.4 % subjects were elders with ethnic minority. Almost 74.3 % of the subjects were married persons.

**Table 1:** Background of study subjects

Item	Category	Number	(%)
Gender	Male	535	40.6
	Female	783	59.4
Education background	Elementary school and below	418	31.8
	Primary school	324	24.6
	High school	251	19.0
	≥College	325	24.6
Ethnicity	Han	1186	90.0
	Ethnic minority	45	3.4
	Missing	87	6.6
Age	60–69	567	43
	70–79	553	42
	>80	198	15.0
Marital status	Married	980	74.3
	Widowed	263	20.0
	Divorced	70	5.3
	Single	5	0.4
Total		1318	

### Principal components analysis

Kaiser–Meyer–Olkin measure of sampling adequacy was 0.954, which indicates adequate sample size for the factor analysis. Bartlett’s test of sphericity was significant (χ^2^ = 1.45E4, *df* = 190,* P* < 0.001). Principal components analysis with varimax rotation solution showed four components with an eigenvalue greater than one which accounted for 59.384 % of total variance. Three factors were extracted from the scale. The eigenvalues and explained % of the variance are shown in Table 2. The reliability of the GAI-CV assessed by Cronbach’s α was 0.937.

**Table 2:** Principal component factor analysis with varimax rotation (eigenvalue >1 and factor loadings >0.5.)

	Mean	SD	Mental anxiety	Negative anxiety	Physical anxiety
1 I worry a lot of the time	0.15	0.354	0.478		
2 I find it difficult to make a decision	0.16	0.369	0.430		
3 I often feel jumpy	0.09	0.283	0.728		
4 I find it hard to relax	0.11	0.318	0.741		
5 I often cannot enjoy things because of my worries	0.08	0.277	0.605		
6 Little things bother me a lot	0.17	0.375	0.581		
7 I often feel like I have butterflies in my stomach	0.12	0.326	0.680		
9 I cannot help worrying about even trivial thing	0.18	0.380	0.601		
10 I often feel nervous	0.11	0.307	0.712		
11 My own thoughts often make me anxious	0.13	0.335	0.670		
13 I think of myself as a nervous person	0.11	0.317	0.592		
20 I often feel upset	0.11	0.318	0.559		
14 I always anticipate the worst will happen	0.07	0.261		0.633	
15 I often feel shaky inside	0.05	0.227		0.739	
16 I think that my worries interfere with my life	0.08	0.274		0.645	
17 My worries often overwhelm me	0.03	0.184		0.755	
19 I miss out on things because I worry too much	0.06	0.239		0.674	
8 I think of myself as a worrier	0.18	0.387			0.604
12 I get an upset stomach due to my worrying	0.13	0.334			0.722
18 I sometimes feel a great knot in my stomach	0.06	0.232			0.595
Eigenvalue			4.676	1.260	1.206

See Table 2, component 1 accounted for 38.97 % of the variance and included twelve items, all pertaining to mental descriptions and including a variety of mental feelings. Therefore, this component was labeled mental anxiety. Cronbach’s α for this component was* α* = 0. 917. Component two accounted for 10.503 % of the variance and comprised five items. All items featured GAI-CV as the negative ideas will result in poor behavior if subjects could not control themselves. All items on this component were negatively phrased, so that a higher score on each item indicated, e.g., an increase in negative ideas. Given the emphasis on anxiety, this component was named anticipation anxiety. Cronbach’s α forth reliability for scale was *α* = 0.851. Lastly, component three accounted for 10.049 % of the variance and consisted of four items. All items referred to the physical response to the anxiety. This was the only component to include negative items. The component was named physical anxiety. Internal consistency was *α* = 0.651. Hence, internal consistency of the three components was good to excellent.

### Confirmatory factor analysis

**Fig. 1:**
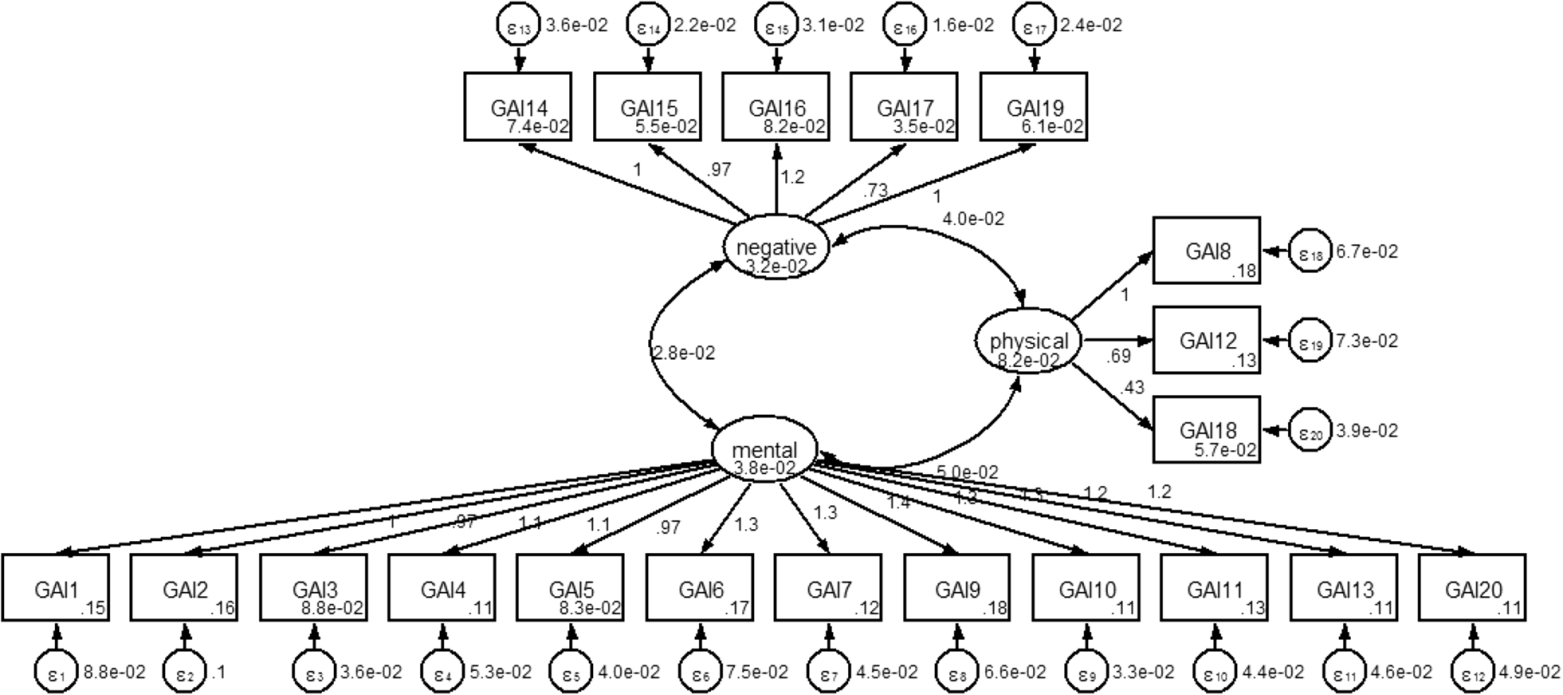
Confirmatory factor analysis

The CFA for the 20-items yielded a three-factor model that fitted the data very well as shown in Fig. 1. All-fit indices of the model had a satisfactory goodness of fit (AIC = −1532.605, BIC = −1206.212, CFI = 0.891, TLI = 0.876, RMSEA = 0.084, SRMR = 0.044, CD = 0.986, and *df* = 63). The results are that the model fits the data good, and is almost certainly the true model for the relations among those indicators. Moreover, CD is 0.986, which provides in percentage form the amount of predictability in the model.

## Discussion

The objectives of this research were to translate the GAI into the Chinese language (GAI-CV) and to determine the factorial structure and validity of the GAI-CV among a group of elderly Chinese adults. To our knowledge, this is the first attempt to validate the GAI-CV in the old Chinese adults. As a result, the final validated Chinese version of the GAI-CV comprises three subscales: mental anxiety, negative anxiety, and physical anxiety. The first subscale, composed of 12 items, assesses mental health of old adults regarding the results obtained with adopting the behaviors listed, as well as the importance of the elder’s perception that others approve or disapprove of performing the behavior. The second subscale, comprising five items, assesses the negative imagines of old adults to identify motives and behaviors related to the mental disorder. Finally, the third subscale, including three items, assesses physical situations of old adults that require of elderly adults decision-making outside their home.

The internal consistency of the GAI-CV was satisfactory. It should be mentioned, however, that the reliability coefficient of the physical anxiety subscale of the Chinese version was below acceptable limits (Cronbach’s α = 0.651). This finding is not very surprising given the fact that this GAI-CV subscale only consists of three items referring to situations.

A similar study of factor analytic study reveals that persons who exhibited symptoms of anxiety produce more factors. A sample of patients with cognitive impairment revealed a four-factor structure of the GAI through principal components analysis [15]. The difference of factor structure between that of Diefenbach et al. (2014) and mine may lie in that there exists healthy elderly persons in my subjectives.

Other factor analytic studies of Geriatric Anxiety Inventory-Short Form (GAI-SF) also showed the best fit for factor structure. For example, the psychometric properties of a Portuguese version of the GAI-SF showed good internal consistency (*α* = 0.77), good convergent, and discriminant validity (*P* < 0.05). The factorial structure presented a single factor that explained 52 % of the variance. The model showed a good fit to the data (χ^2^ = 1.233, TLI = 0.997, CFI = 0.999, RMSEA = 0.020) [16]. It suggests that GAI-SF may be used as an alternative for GAI-CV.

The factor structure from this study is guessed to be generalized to the whole population in China. Because another study indicates that the GAI and Geriatric Anxiety Scale may be good alternatives to anxiety measures not designed specifically for older adults [17].

Complementally, this study suggests anxiety may come from internal motivation besides shame [18] and empty-nest living arrangement [19]. Thus, upgrade of life attitude may reduce the harm from anxiety prevalence among old population in China.

The findings from this study indicated that improving quality of life in this age group becomes a very important task. Early studies show burnout management [20], mental health tutorship [13], Tai chi [21, 22], and morita therapy [23] should be considered in the city of Beijing. To effectively reduce the anxiety symptoms of the elderly population, this study’s finding in addition to those physical activities highlights prevention from mental breakdown.

## Conclusions

The results of this study suggest that the GAI-CV is a valid and reliable tool for measuring anxiety of the elderly adults aged 60 years and above in China Beijing. Based on this study, we can guess anxiety consists of psychological, negative, and physical dimensions. The internal consistency of the GAI-CV scales was found to be satisfactory. Exploratory factor analysis yielded a multiple-factor model, and a CFA demonstrated reasonable fit for a three-factor correlated model in the total sample. Therefore, it can be concluded that the GAI-CV seems to be a promising scale for assessing various aspects of anxiety that is suitable for the elderly adults in China.

The results from China Beijing context suggest GAI-CV has been not only used in studies of Western countries but also applied in the Chinese population. Moreover, the factor analysis shows GAI-CV may be reliable and valid to measure anxiety in other developing countries.

Several limitations of the present study should be noted. A first limitation pertains to normal, psychologically healthy elders, and so the reliability and validity of the GAI-CV in clinically referred elders remains to be established. Second, a number of psychometric properties of the GAI-CV remain to be tested. The current study did not examine the test-retest reliability of the GAI-CV, and also the predictive, convergent, divergent, and discriminant validity of the scale need to be investigated.
